# Symptom presentation by phenotype of postural orthostatic tachycardia syndrome

**DOI:** 10.1038/s41598-023-50886-8

**Published:** 2024-01-02

**Authors:** Allison M. Angeli, Bradley R. Salonen, Ravindra Ganesh, Ryan T. Hurt, Ahmed Abdalrhim, Michael Mueller, Mary Volcheck, Christopher Aakre

**Affiliations:** 1 Internal Medicine Residency Program, Mayo Clinic, Rochester, MN USA; 2 Department of General Internal Medicine, Mayo Clinic, Rochester, MN USA

**Keywords:** Signs and symptoms, Cardiovascular diseases, Neurological disorders

## Abstract

Postural orthostatic tachycardia syndrome (POTS) presents heterogeneously and is diagnosed when appropriate symptoms are present in conjunction with a heart rate increase of at least 30 beats-per-minute upon standing without orthostatic hypotension. Much of the current understanding of POTS is based on clinical expertise, particularly regarding POTS phenotypes and their potential role in targeting pharmacologic treatment. This study describes the symptom presentation of POTS by phenotypes at a subspecialty POTS clinic. Data was collected prospectively during clinical visits between April 17, 2014 and February 8, 2021. This data was abstracted retrospectively by chart review. Most of the 378 study participants were female (89.9%) with a mean age 23.0 ± 4.9 years. Lightheadedness was the most common (97.6%) symptom and the most disruptive of quality of life (29.9%). Patients reported substantial functional impairment across multiple life domains, with 3.0 ± 2.8 days lost and 4.7 ± 2.3 unproductive days per week. There were no differences in symptom presentation among POTS phenotypes. POTS phenotypes are not distinguishable based on symptoms alone; if phenotyping is sought, testing is necessary. Further research is needed in better classifying POTS phenotypes with the potential goal of tailoring treatment.

## Introduction

Postural orthostatic tachycardia syndrome (POTS) is a condition that presents heterogeneously. Common symptoms include lightheadedness, presyncope, palpitations, fatigue, difficulty sleeping, difficulty concentrating, headache, nausea, and more^1–4^. There have been many studies which formally assess clinical characteristics of POTS, but these are often limited by small sample size, non-generalizability, or an unconfirmed diagnosis^4–7^. Much of the current understanding of the presentation of POTS comes from clinical expertise.

POTS is defined by a 30 beat-per-minute (bpm) or more increase in heart rate within 10 min upon upright tilt, in the absence of orthostatic hypotension^1,2,8^. This may be diagnosed by an active stand test using a sphygmomanometer, or a head-up-tilt test (HUTT). Associated symptoms must be present as well to meet the diagnosis. Prior to making a diagnosis of POTS, an evaluation to rule out other etiologies with overlapping symptoms, such as structural heart problems, should be completed^8^. Patients may undergo other supporting tests in order to better define the underlying pathophysiology.

POTS may be classified into phenotypes—neuropathic, hypovolemic, and hyperadrenergic—based on suspected pathophysiologic mechanism. Neuropathic POTS involves impaired peripheral vasoconstriction and excessive blood pooling below the waist via impaired small fiber nerve function^1,3,9^. Hypovolemic POTS is due to dysfunction of the renin–angiotensin–aldosterone system resulting in low plasma blood volume and may be identified with 24-h urine sodium concentration less than 100 mmol/L^1^. Hyperadrenergic POTS is characterized by high central sympathetic drive, evident by an increase in systolic blood pressure (SBP) on HUTT and a standing plasma norepinephrine level of 600 pg/mL or more^1,2,9^. These three phenotypes are not mutually exclusive and may be present in any combination in patients with POTS^1,3^. Other medical conditions, such as joint hypermobility, have been hypothesized to produce physiologies that can mimic or contribute to these phenotypes. For example, more compliant connective tissue in joint hypermobility syndromes may result in more venous pooling in the lower extremities and low effective intrathoracic volume^9,10^.

Phenotype determination in POTS patients is sought in an effort to tailor treatment based on underlying pathophysiology. Nonpharmacologic interventions are typically considered first-line treatments, followed by pharmacologic treatments for refractory symptoms^7,9^. For hypovolemic POTS, interventions are aimed at increasing plasma volume, including but not limited to high fluid and salt intake, exercise, and fludrocortisone^3^. In neuropathic POTS, treatments target increasing venous return either by external compression such as compression stockings or lower extremity countermaneuvers or by pharmacologic vasoconstriction such as with midodrine^3^. Lastly, in hyperadrenergic POTS, measures are directed at dampening sympathetic output including avoidance of sympathetic agents such as serotonin-norepinephrine reuptake inhibitors, as well as utilizing pharmacotherapy such as beta-blockers^3,9^. These phenotypes are not mutually exclusive and all lead to the symptoms experienced by patients in POTS^1,3,9^. However, the treatment response in POTS is highly heterogenous. Little is understood about the prevalence of POTS phenotypes, and the implication, if any, of using symptoms to assign phenotype(s) and guide initial treatment. Moreover, to our knowledge, there is no data on the prevalence and clinical presentations of overlapping POTS phenotypes. This study sought to describe the clinical presentation of POTS patients evaluated at a specialty POTS clinic, focusing on evaluating symptom differences among phenotypes.

## Methods

Study protocol was reviewed and deemed exempt by the Mayo Clinic Institutional Review Board (IRB#21-000572). All methods were carried out in accordance with relevant guidelines and regulations. Informed consent was obtained from all subjects and/or their legal guardians. Patients without research authorization were excluded from analysis.

All patients were seen by general internists in the Integrative Medicine POTS Clinic at Mayo Clinic in Rochester, Minnesota from April 17, 2014 to February 8, 2021. Patients were referred from multiple sources including internal medicine, specialty practice, and self-referral. All patients underwent a standardized pre-visit clinical nursing assessment. During the clinical assessment, the following information was collected: demographics, relationship status, occupation, tobacco use history, previous POTS diagnosis (and age, if applicable), comorbid symptoms, top symptoms that disrupt quality of life, and self-reported disability (Sheehan Disability Scale). Patients underwent a standardized testing protocol to confirm the diagnosis of POTS (head up tilt) and specific laboratory testing to establish the POTS phenotype(s) to guide therapy. All patients underwent our autonomic reflex screen which includes Quantitative Sudomotor Axon Reflex Testing (QSART), heart rate response to deep breathing, and HUTT^11^. Abnormal QSART was reporter-dependent and not standardized. Abnormal QSART was followed up with thermoregulatory sweat testing (TST) when indicated. Further phenotypic testing included supine and standing catecholamines and a 24-h urine sodium. Of note, the catecholamine blood draws were obtained at a specialized endocrine testing center with the first blood draw being supine and the second blood draw being after 10 min of standing. Testing was not performed if done in the last 6 months and results were available for review. Certain laboratory tests were not performed if not indicated by physician judgment after initial assessment. Any medications that could potentially interfere with testing were recommended to be discontinued, however this was not possible in all patients. Phenotypes were determined as hypovolemic for urine sodium less than 100 mmol/24 h, neuropathic for abnormal sudomotor response or abnormal sweat pattern on TST, and hyperadrenergic for standing norepinephrine > 600 pg/mL or an increase in either systolic or diastolic blood pressure of ≥ 10 mm Hg on HUTT^1,2,9,12^.

Data was collected prospectively during clinical visits through standardized nursing documentation and abstracted retrospectively by chart review. Statistical analysis was performed with R 3.6.3. Only patients with a prior diagnosis or diagnosed with POTS during this evaluation were included in statistical analysis. Descriptive statistics were analyzed by frequencies and means with standard deviations. ANOVA or chi-squared tests were used to compare clinical phenotypes for continuous and categorical data, respectively. Due to multiple comparisons, Bonferroni correction was utilized to adjust the level of significance to reflect the number of comparisons.

## Results

### Study eligibility

Of the 430 participants, 378 were previously diagnosed with POTS or were found to meet diagnostic criteria and included in this study.

### Demographics

Of the 378 participants, few did not self-report specific demographic information or did not have the information available on chart review, resulting in a variable N. Most of the study participants were female (89.9%, 340/378), mean age 23.0 ± 4.9 years-old at time of study participation (n = 378), single (68.3%, 258/378), either a student (38.5%, 145/377) or employed (35.0%, 132/377) and did not smoke tobacco (91.1%, 336/369). Body mass index varied (24.8 ± 6.0 kg/m^2^, n = 369). The majority had a prior diagnosis of POTS (81.2%, 306/377), and the mean age of initial diagnosis was 21.0 ± 5.6 years-old (n = 299) Table 1.

**Table 1:** Demographics.

	N	Frequency (%)	Mean ± SD
Prior diagnosis of POTS	306/377	81.2	–
Sex			
Female	340/378	89.9	–
Male	38/378	10.1	–
Age			
At diagnosis of POTS	299	–	21.0 ± 5.6
At consult	378	–	23.0 ± 4.9
BMI	369	–	24.8 ± 6.0
Relationship			
Single	258/378	68.3	–
Committed Relationship	62/378	16.4	–
Married	54/378	14.3	–
Divorced	4/378	1.1	–
Occupation			
Employed	132/377	35.0	–
Unemployed	70/377	18.6	–
Disabled	10/377	2.7	–
Homemaker	7/377	1.9	–
Student	145/377	38.5	–
Other	13/377	3.4	–
Tobacco use	33/369	8.9	

N varied as some specific demographic information was not self-reported in few of the 378 participants.

### Phenotypes

Hyperadrenergic POTS was the most common phenotype (75.0%, 264/352), followed by hypovolemic POTS (44.9%, 158/352) and neuropathic POTS (37.8%, 133/352). These were not mutually exclusive, and many patients had a combination of phenotypes with 41.7% (147/352) having two phenotypes, and 11.4% (40/352) with a contribution from all three Table 2.

**Table 2:** POTS phenotypes.

Phenotype*	N = 352 †	Frequency (%)
A	93	26.4
AO	81	23.0
AN	50	14.2
ANO	40	11.4
N	27	7.7
None	24	6.8
O	21	6.0
NO	16	4.5

*A = Hyperadrenergic, N = Neuropathic, O = Hypovolemic.

†Phenotypes unable to be determined in 26 participants due to incomplete testing.

### Symptoms

The most common symptom was lightheadedness (97.6%, 369/378), followed by rapid heart rate, headache, dizziness, cognitive difficulties, weakness, vision changes, exercise intolerance, palpitations, shortness of breath, chest pain, fatigue upon standing, anxiety and tremulousness—all experienced by over 2/3 of participants [Supplementary Table S1]. Of the reported symptoms, the top two that most disrupt the quality of life were lightheadedness (29.9%, 113/378) and dizziness (28.0%, 106/378), followed by fatigue upon standing, fatigue, headaches, and cognitive difficulties [Supplementary Table S2]. Other common chronic symptoms reported include headaches (60.3%, 228/378), pain (52.1%, 197/378), nausea (50.8%, 192/378) and constipation (25.9%, 98/378) [Supplementary Table S3]. Ongoing gastrointestinal symptoms were very common (81.2%, 307/378). ANOVA was performed comparing phenotypes for the frequencies of each symptom reported [Supplementary Tables S1–S3]. Bonferroni correction was utilized to adjust the level of significance in order to reduce the probability of a type I error. There were no statistical differences in any symptoms across phenotypes [Supplementary Tables S1–S3].

### Diagnostics

**Figure 1:**
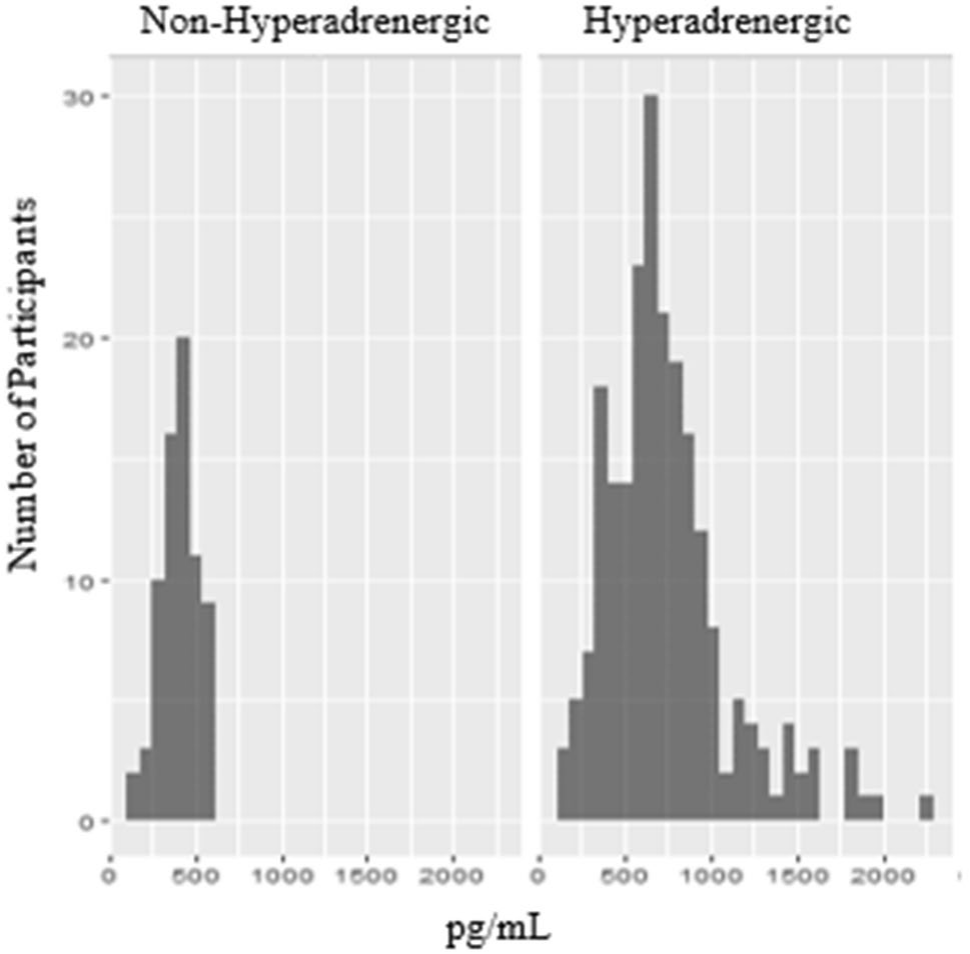
Distribution of standing serum catecholamines in hyperadrenergic versus non-hyperadrenergic POTS. The standing norepinephrine was completed in all 378 participants, with a mean of 744 ± 359 pg/mL in all those with hyperadrenergic POTS (n = 264) and 415 ± 102 pg/mL in those without hyperadrenergic POTS (n = 71). The means and distribution suggest that the serum standing serum norepinephrine cut-off of 600 pg/mL may serve better to rule-out hyperadrenergic POTS rather than rule-in.

On the head-up tilt test, the mean supine heart rate was 75.7 ± 13.8 bpm and mean highest heart rate was 109.2 ± 18.7 bpm, with a mean change in heart rate of 33.5 ± 14.4 bpm [Supplementary Table S4]. The standing norepinephrine was completed in all 378 participants, with a mean of 744 ± 359 pg/mL in all those with hyperadrenergic POTS (n = 264) and 415 ± 102 pg/mL in those without hyperadrenergic POTS (n = 71) [Fig. 1]. The 24-h urine sodium was completed in 355 participants, with a mean of 119.2 ± 61.2 mmol/24 h in all (n = 355), 69.7 ± 22 in those with hypovolemic POTS (n = 158), and 158.9 ± 53 in those without hypovolemic POTS (n = 197). QSART was completed in 367 participants, of which 82 (22.3%, 82/367) were abnormal. TST was completed in 276 participants, of which 95 (34.4%, 95/276) were abnormal.

### Disability

The average Sheehan Disability Scale was consistent with moderate to marked functional impairment in all three domains—work/school (7.1 ± 2.8), social life (7.0 ± 2.5), and family life/home responsibilities (6.4 ± 2.8). The average number of days in the past week during which one was unable to carry out responsibilities due to symptoms was 3.0 ± 2.8 days. The average number of days in the past week that symptoms reduced productivity at school or work was 4.7 ± 2.3 days. There were no statistically significant differences among phenotypes [Supplementary Table S5].

## Discussion

Much of the current knowledge on POTS is based on clinical practice. In particular, little is understood about the prevalence of POTS phenotypes, and the implication, if any, of using symptoms to assign phenotype(s) and guide initial treatment. There are few studies that do compare clinical presentation between POTS phenotypes, and, to our knowledge, there are no studies that compare overlapping phenotypes. The present study was a large sample size (n = 378) describing the clinical presentation of POTS patients at a specialty POTS clinic, with a focus on evaluating symptom presentation across phenotypes.

This study demonstrated a patient demographic consistent with prior literature—young-adult, females who are either students or employed^4,5,8^. Notably, this did not distinguish between full- and part-time work/studies, or whether patients required disability accommodations, as this information was not available at time of chart review. It is likely that many of these patients were part-time, did online school, and/or required disability accommodations, or that this represents sample bias related to the rigors of presenting to a destination medical center. Marital status has not been well-described in current literature; most patients in our study were single, likely due to age and associated life phase.

The large variety of symptoms reported are consistent with the previously recognized heterogeneous presentation of POTS. Lightheadedness was the most common symptom and top disruptor of quality of life. Other common symptoms included rapid heart rate, headaches, dizziness, cognitive difficulties, weakness, vision changes, palpitations, exercise intolerance, shortness of breath, chest pain, and anxiety. Some common symptoms, such as fatigue, were not specifically assessed though were reported by some under “other symptoms” owing to the lower prevalence of 9.5% in this study. The lower prevalence may also be due to our clinic’s protocol, as patients presenting to our healthcare system with fatigue-predominance are directed to the Myalgic Encephalomyelitis/Chronic Fatigue Syndrome (ME/CFS) clinic. Indeed, orthostatic intolerance is one of the diagnostic criteria for ME/CFS under the 2015 Institute of Medicine criteria^13^.

There were no statistically significant differences in symptoms among phenotypes. This substantiates POTS as one syndrome, though would not negate distinct and non-mutually exclusive pathophysiologic phenotypes, as the etiology of POTS is thought to be multifactorial. However, the significant phenotype overlap in this study may have masked potential differences. There are three commonly thought of pathophysiological phenotypes—hypovolemic, hyperadrenergic, and neuropathic. There is some uncertainty in the field in regard to the three distinct phenotypes, as well as their tests and associated cut-off values for classification. This study is one piece of ongoing research in those areas. Current evidence and clinical expertise suggest POTS phenotypes may be determined with 24-h urine sodium for hypovolemic, standing plasma norepinephrine or increase in systolic blood pressure on HUTT for hyperadrenergic, and either skin biopsy or QSART followed by TST for neuropathic^1–3,9^.

Phenotypic prevalence is not yet widely reported in current literature. Compared to a cohort study of 152 participants by Thieben et al.^11^, our study demonstrated neuropathic POTS to be less common despite more inclusive criteria (37.8% on QSART or TST versus 42.8% on QSART and 53.8% on TST), potentially due to referral bias to Neurology rather than our POTS clinic. Additionally, in comparison to Thieben et al.^11^ our study showed hypovolemic POTS to be more common with the same criteria (44.9% vs. 28.9%), and hyperadrenergic POTS to be more common as expected with more inclusive criteria (75.0% vs. 29%). Other studies and subspecialty centers have utilized skin biopsy to assess intraepidermal nerve fiber density for small fiber neuropathy and/or neuropathic phenotypes^14–16^. Skin biopsy is more readily available and cost-effective outside of specialty centers and can be utilized to evaluate for neuropathic POTS. Skin biopsy is not routinely performed at our clinic, so QSART and TST were relied on for phenotyping neuropathic POTS. The rate of abnormal QSART testing in our cohort was lower (22.3%) compared to the existing data wherein about 30–50% of patients with POTS are found to have abnormal QSART testing and may represent differences in sensitivity and specificity between techniques^16,17^.

Our study additionally describes pure phenotype and crossover phenotype groups, with a purely hyperadrenergic phenotype as the most common, followed by hyperadrenergic crossover phenotypes. Of note, 6.8% (n = 24) did not fall into any of the existing phenotypes, suggesting either additional mechanisms by which POTS may develop or potentially too rigorous criteria for phenotypes. For example, the current serum standing norepinephrine cut-off of 600 pg/mL may serve better to rule-out hyperadrenergic POTS based on the means and standard deviations identified in this study (Fig. 1).

POTS phenotypes are typically sought in an effort to tailor treatment, often after first-line nonpharmacologic measures have not provided significant improvement in symptoms and quality of life. For example, for hyperadrenergic POTS, the sympathetic output is blocked via beta-blockers, or sympathetic symptoms such as tachycardia may be combated by pharmacotherapies such ivabradine which inhibits I(f) channels. In neuropathic POTS, pharmacotherapy targets vasoconstriction with agents such as pyridostigmine and midodrine. Increasing plasma volume is sought with agents such as fludrocortisone for hypovolemic POTS^3,9^. High quality studies, such as randomized control trials, have not yet been done for these pharmacotherapies. As such, the use of these medications is based on clinical expertise and experience.

POTS impacts quality of life to a degree consistent with that of chronic obstructive pulmonary disease and heart failure^18^. Functional impairment secondary to POTS-related symptoms were assessed in this study using The Sheehan Disability Scale (SDS). The SDS is a survey that evaluates the severity of disability in three domains of work/school, family life/home responsibilities, and social life/leisure activities. Two additional questions on days lost and days unproductive in the last week due to symptoms are also included. Prior studies assessing the SDS have shown reliability, validity, and high internal consistency^19–21^. Results of the SDS in our study demonstrated moderate to marked functional impairment across multiple life domains with multiple days per week impacted by the ability to be productive and complete responsibilities. There were no differences among phenotypes. Despite this, only 2.7% of patients self-reported their occupation as “disabled”, which is far lower than the 34.2% reported in a large survey study^22^. As discussed above, our lower numbers may reflect sampling bias due to the rigors and costs of presenting to a destination medical center while concomitantly the survey data likely overestimated the prevalence of disability.

Some data in this study was collected via survey and interview, with inherent self-report bias. All patients were seen at a subspecialty clinic at a destination medical center that is well-known in the field of POTS, and thus may only capture a subset of the general POTS population. In fact, most of our patients had symptoms for an average of 2 years before they were evaluated by our clinic and had not improved with typical non-pharmacologic conservative management strategies which largely target increasing venous return and increasing plasma volume—neuropathic and hypovolemic POTS, respectively^3^. Thus, our clinic may see more hyperadrenergic POTS patients. Not all participants completed all the phenotype screening tests, and thus, the results may not be fully representative. However, as these tests were discontinued by an evaluating POTS specialist often based on the tests being performed previously, the likelihood of them being clinically significant is low. This was done in an effort to provide cost-conscious care, particularly given the high economic burden on patients with POTS^22^.

## Conclusion

In line with current literature, this study demonstrates that POTS often occurs in young-adult females with a wide variety of symptoms that cause functional impairment in multiple domains of life. Symptoms had no differences across phenotypes, supporting POTS as a unified syndrome with a multifactorial etiology. As phenotypes cannot be distinguished by symptoms alone, clinicians should seek further testing if phenotyping is pursued, including a 24-h urine sodium for hypovolemic POTS, supine and standing catecholamines and SBP rise on HUTT for hyperadrenergic POTS, and skin biopsy or QSART with reflex to TST for neuropathic POTS. These tests should be considered in patients whose symptoms are refractory to the first line nonpharmacologic treatment strategies, in order to trial pharmacologic treatment aimed at underlying phenotype(s). Notably, the lack of difference in symptoms among phenotypes in this study could be due to the high proportion of phenotype overlap, the lack of sufficiently sensitive and specific tests for phenotypes, as well as currently unknown and/or missed phenotypes due to an incomplete understanding of the pathophysiologic mechanisms by which POTS develops. Future research should be aimed at these measures, to achieve better phenotyping, and in turn potentially tailor treatment.

### Supplementary Information

Supplementary Tables.

## Data Availability

The data that support the findings of this study are available from the corresponding author upon reasonable request.
