# Supplementary material for: Symptom presentation by phenotype of postural orthostatic tachycardia syndrome
Source: Sci Rep. 2024 Jan 2;14:205. doi: 10.1038/s41598-023-50886-8 (PMC10761725; doi:10.1038/s41598-023-50886-8)
Supplement: Supplementary file 1 — Supplementary Tables. [file 41598_2023_50886_MOESM1_ESM.docx]

**Supplemental Material for**

**Symptom Presentation by Phenotype of Postural Orthostatic Tachycardia Syndrome**

**Allison M. Angeli MD^1^*, Bradley R. Salonen MD^2^, Ravindra Ganesh MBBS MD^2^, Ryan T. Hurt MD PhD^2^, Ahmed Abdalrhim MD^2^, Michael Mueller MD^2^, Mary Volcheck MSN RN, Christopher Aakre MD^2^**

^1^Internal Medicine Residency Program, Mayo Clinic, Rochester, MN

^2^Department of General Internal Medicine, Mayo Clinic, Rochester, MN

**Supplementary Table S1. Symptom Prevalence by POTS Phenotype**

|  | **Frequency (%)** | | | | | | | | | |  |
| --- | --- | --- | --- | --- | --- | --- | --- | --- | --- | --- | --- |
|  | **All** | **Pure A** | **Pure N** | **Pure O** | **AN** | **AO** | **NO** | **ANO** | **None** | | **P-value** |
| Sample Size | 378 | 93 | 27 | 21 | 50 | 81 | 16 | 40 | 24 | |  |
| Lightheadedness | 97.6 | 97.8 | 100.0 | 100.0 | 96.0 | 98.8 | 93.8 | 100.0 | 91.7 |  | 0.32 |
| Rapid Heart Rate | 90.7 | 90.3 | 92.6 | 85.7 | 90.0 | 87.7 | 100.0 | 97.5 | 87.5 |  | 0.57 |
| Headache | 89.7 | 89.2 | 88.9 | 76.2 | 90.0 | 90.1 | 87.5 | 95.0 | 91.7 |  | 0.61 |
| Dizziness | 89.7 | 87.1 | 88.9 | 95.2 | 84.0 | 91.4 | 100.0 | 90.0 | 91.7 |  | 0.64 |
| Cognitive Difficulties | 85.7 | 84.9 | 85.2 | 81.0 | 88.0 | 86.4 | 81.2 | 87.5 | 87.5 |  | 0.99 |
| Weakness | 83.9 | 80.6 | 85.2 | 71.4 | 80.0 | 88.9 | 93.8 | 92.5 | 79.2 |  | 0.31 |
| Vision Changes | 81.0 | 83.9 | 81.5 | 81.0 | 88.0 | 84.0 | 75.0 | 72.5 | 75.0 |  | 0.77 |
| Exercise Intolerance | 80.7 | 81.7 | 63.0 | 66.7 | 84.0 | 80.2 | 81.2 | 90.0 | 87.5 |  | 0.05 |
| Palpitations | 80.2 | 77.4 | 81.5 | 81.0 | 78.0 | 84.0 | 81.2 | 90.0 | 79.2 |  | 0.82 |
| Shortness Of Breath | 77.2 | 73.1 | 66.7 | 76.2 | 82.0 | 81.5 | 75.0 | 82.5 | 70.8 |  | 0.70 |
| Chest Pain | 70.6 | 68.8 | 77.8 | 61.9 | 82.0 | 64.2 | 75.0 | 65.0 | 75.0 |  | 0.27 |
| Fatigue Upon Standing | 68.3 | 69.9 | 51.9 | 57.1 | 66.0 | 75.3 | 81.2 | 60.0 | 75.0 |  | 0.21 |
| Anxiety | 68.0 | 62.4 | 70.4 | 71.4 | 72.0 | 70.4 | 68.8 | 72.5 | 75.0 |  | 0.91 |
| Tremulousness | 67.7 | 63.4 | 59.3 | 61.9 | 68.0 | 71.6 | 62.5 | 77.5 | 75.0 |  | 0.71 |
| Fainting Or Passing Out | 56.6 | 61.3 | 55.6 | 57.1 | 52.0 | 48.1 | 68.8 | 52.5 | 62.5 |  | 0.69 |
| New Or Worsening Motion Sickness | 41.3 | 34.4 | 44.4 | 33.3 | 48.0 | 50.6 | 37.5 | 40.0 | 33.3 |  | 0.58 |
| Orthostatic Headaches | 38.6 | 36.6 | 37.0 | 28.6 | 40.0 | 46.9 | 12.5 | 40.0 | 37.5 |  | 0.32 |
| Other* | 31.5 | 32.4 | 18.5 | 42.9 | 30 | 28.4 | 12.4 | 50 | 20.9 |  |  |
| Fatigue | 9.5 | 5.4 | 7.4 | 9.5 | 14.0 | 9.9 | 0.0 | 15.0 | 0.0 |  | 0.24 |
| Other             Remainder**†** | 7.4 | 8.6 | 3.7 | 19.0 | 6.0 | 7.4 | 6.2 | 7.5 | 4.2 |  | 0.63 |
| Paresthesia | 5.0 | 6.5 | 0.0 | 4.8 | 4.0 | 6.2 | 6.2 | 7.5 | 4.2 |  | 0.93 |
| Heat Or Cold             Intolerance | 4.8 | 5.4 | 3.7 | 4.8 | 2.0 | 4.9 | 0.0 | 7.5 | 8.3 |  | 0.88 |
| Sweating | 4.8 | 6.5 | 3.7 | 4.8 | 4.0 | 0.0 | 0.0 | 12.5 | 4.2 |  | 0.13 |
| Lightheadedness As a Headache Trigger | 28.3 | 35.5 | 25.9 | 23.8 | 22.0 | 28.4 | 31.2 | 27.5 | 25.0 |  | 0.81 |
| Pelvic pain | 1.1 | 2.2 | 0.0 | 0.0 | 0.0 | 0.0 | 0.0 | 2.5 | 4.2 |  | 0.58 |

Symptoms experienced were self-reported among participants with POTS and were analyzed via ANOVA among phenotypes. Using Bonferroni correction, statistical significance was calculated to be p < 0.0021. There were no symptoms that were statistically significant among phenotypes.

* subgroup symptoms were reported in free text under “Other” at > 4.0% frequency

† additional reported symptoms under “Other” at < 4.0% frequency

A = Hyperadrenergic, N = Neuropathic, O = Hypovolemic

**Supplementary Table S2. Top Two Symptoms that Disrupt Quality of Life by POTS Phenotype**

|  | **Frequency (%)** | | | | | | | | |  |
| --- | --- | --- | --- | --- | --- | --- | --- | --- | --- | --- |
|  | **All** | **Pure A** | **Pure N** | **Pure O** | **AN** | **AO** | **NO** | **ANO** | **None** | **P-value** |
| Sample Size | 378 | 93 | 27 | 21 | 50 | 81 | 16 | 40 | 24 |  |
| Other**†** | 67.5 | 62.7 | 66.6 | 66.8 | 80 | 70.3 | 37.2 | 82.5 | 54.3 | N/A |
| Other Remainder**‡** | 15.6 | 10.8 | 22.2 | 4.8 | 20.0 | 14.8 | 6.2 | 25.0 | 16.7 | 0.25 |
| Pain | 14.6 | 14.0 | 3.7 | 33.3 | 20.0 | 13.6 | 6.2 | 12.5 | 4.2 | 0.07 |
| Nausea | 13.2 | 14.0 | 7.4 | 14.3 | 18.0 | 16.0 | 6.2 | 7.5 | 16.7 | 0.74 |
| GI Symptoms**§** | 7.4 | 6.5 | 18.5 | 0.0 | 6.0 | 6.2 | 0.0 | 12.5 | 8.3 | 0.20 |
| Vision Changes | 7.4 | 10.8 | 3.7 | 4.8 | 6.0 | 6.2 | 6.2 | 12.5 | 4.2 | 0.74 |
| Heat Or Cold         Intolerance | 3.4 | 1.1 | 3.7 | 4.8 | 4.0 | 6.2 | 0.0 | 5.0 | 4.2 | 0.77 |
| Insomnia | 2.4 | 2.2 | 0.0 | 4.8 | 2.0 | 4.9 | 6.2 | 0.0 | 0.0 | 0.58 |
| All Symptoms         Combined | 1.9 | 1.1 | 7.4 | 0.0 | 2.0 | 1.2 | 6.2 | 2.5 | 0.0 | 0.41 |
| Increased         Sweating | 1.6 | 2.2 | 0.0 | 0.0 | 2.0 | 1.2 | 0.0 | 5.0 | 0.0 | 0.74 |
| Lightheadedness | 29.9 | 33.3 | 29.6 | 28.6 | 28.0 | 32.1 | 31.2 | 37.5 | 16.7 | 0.82 |
| Dizziness | 28.0 | 24.7 | 22.2 | 33.3 | 22.0 | 34.6 | 43.8 | 27.5 | 33.3 | 0.54 |
| Fatigue Upon Standing | 26.2 | 19.4 | 18.5 | 28.6 | 30.0 | 22.2 | 43.8 | 25.0 | 37.5 | 0.30 |
| Fatigue | 20.6 | 23.7 | 14.8 | 38.1 | 20.0 | 17.3 | 12.5 | 25.0 | 12.5 | 0.37 |
| Headaches | 19.0 | 20.4 | 25.9 | 19.0 | 14.0 | 16.0 | 25.0 | 15.0 | 20.8 | 0.88 |
| Cognitive Difficulties | 18.5 | 23.7 | 22.2 | 14.3 | 12.0 | 17.3 | 18.8 | 15.0 | 16.7 | 0.78 |
| Rapid Heart Rate | 15.1 | 12.9 | 0.0 | 14.3 | 14.0 | 22.2 | 12.5 | 17.5 | 20.8 | 0.24 |
| Fainting Or Passing Out | 12.2 | 16.1 | 7.4 | 9.5 | 8.0 | 11.1 | 12.5 | 17.5 | 12.5 | 0.79 |
| Weakness | 10.6 | 16.1 | 0.0 | 4.8 | 6.0 | 13.6 | 6.2 | 15.0 | 12.5 | 0.23 |
| Shortness of Breath | 9.5 | 8.6 | 11.1 | 14.3 | 10.0 | 11.1 | 0.0 | 10.0 | 8.3 | 0.91 |
| Palpitations | 8.7 | 6.5 | 7.4 | 19.0 | 6.0 | 12.3 | 6.2 | 15.0 | 4.2 | 0.40 |
| Chest Pain | 8.5 | 5.4 | 14.8 | 0.0 | 10.0 | 11.1 | 18.8 | 7.5 | 8.3 | 0.40 |
| Exercise Intolerance | 5.0 | 9.7 | 0.0 | 4.8 | 2.0 | 1.2 | 0.0 | 12.5 | 4.2 | 0.05 |
| Tremulousness | 3.2 | 3.2 | 0.0 | 0.0 | 8.0 | 2.5 | 6.2 | 5.0 | 0.0 | 0.46 |
| Anxiety | 3.2 | 2.2 | 3.7 | 9.5 | 4.0 | 2.5 | 6.2 | 0.0 | 4.2 | 0.62 |

Top two symptoms that disrupted quality of life were self-reported among participants with POTS and were analyzed via ANOVA among phenotypes. Using Bonferroni correction, statistical significance was calculated to be p < 0.0021. There were no symptoms that were statistically significant among phenotypes.

† subgroup symptoms were reported in free text under “Other” at > 1.0% frequency

‡ additional reported symptoms under “Other” at < 1.0% frequency each. These included orthostatic headaches, new or worsening motion sickness, pelvic pain, and lightheadedness as a headache trigger

§ GI symptoms did not include nausea. They did include a wide variety of symptoms: abdominal pain/cramps, vomiting, constipation, diarrhea, heartburn, low appetite, early satiety, gastroparesis, GERD, acid reflux, stomach ulcers, painful defecation, mucus in stool, blood in stool, change in stool characteristics, and rectal bleeding.

A = Hyperadrenergic, N = Neuropathic, O = Hypovolemic

**Supplementary Table S3. Chronic Symptoms by POTS Phenotype**

|  | **Frequency (%)** | | | | | | | | |  |
| --- | --- | --- | --- | --- | --- | --- | --- | --- | --- | --- |
|  | **All** | **Pure A** | **Pure N** | **Pure O** | **AN** | **AO** | **NO** | **ANO** | **None** | **P-value** |
| Sample Size | 378 | 93 | 27 | 21 | 50 | 81 | 16 | 40 | 24 |  |
| Other **†** | 87.6 | 93.7 | 85.1 | 57.2 | 108 | 80 | 49.8 | 105 | 108.3 | N/A |
| Other       Remainder**‡** | 19.3 | 14.0 | 22.2 | 9.5 | 22.0 | 25.9 | 12.5 | 20.0 | 16.7 | 0.51 |
| Hypermobility | 9.5 | 14.0 | 11.1 | 4.8 | 4.0 | 4.9 | 0.0 | 12.5 | 12.5 | 0.23 |
| Sinus       Congestion | 8.7 | 11.8 | 7.4 | 14.3 | 12.0 | 4.9 | 6.2 | 7.5 | 12.5 | 0.75 |
| Urinary       Symptoms**§** | 6.9 | 3.2 | 3.7 | 9.5 | 10.0 | 4.9 | 0.0 | 20.0 | 12.5 | 0.03 |
| Weight Gain       > 10 lbs | 6.1 | 4.3 | 7.4 | 4.8 | 6.0 | 6.2 | 6.2 | 10.0 | 8.3 | 0.97 |
| Cough | 6.1 | 9.7 | 7.4 | 0.0 | 6.0 | 4.9 | 6.2 | 5.0 | 8.3 | 0.82 |
| Unusual Thirst | 5.6 | 6.5 | 3.7 | 0.0 | 8.0 | 7.4 | 0.0 | 7.5 | 0.0 | 0.64 |
| Depressed Mood | 5.6 | 3.2 | 7.4 | 9.5 | 8.0 | 3.7 | 12.5 | 7.5 | 4.2 | 0.71 |
| Joint Swelling | 5.3 | 8.6 | 3.7 | 0.0 | 8.0 | 4.9 | 0.0 | 2.5 | 8.3 | 0.59 |
| Weight Loss       > 10 lbs | 5.3 | 5.4 | 7.4 | 4.8 | 10.0 | 4.9 | 6.2 | 2.5 | 4.2 | 0.89 |
| Tendency to Fall | 5.0 | 6.5 | 3.7 | 0.0 | 8.0 | 3.7 | 0.0 | 5.0 | 12.5 | 0.54 |
| Dysphagia | 4.2 | 6.5 | 0.0 | 0.0 | 6.0 | 3.7 | 0.0 | 5.0 | 8.3 | 0.66 |
| Headaches | 60.3 | 57.0 | 44.4 | 38.1 | 70.0 | 63.0 | 62.5 | 70.0 | 58.3 | 0.12 |
| Pain | 52.1 | 51.6 | 33.3 | 47.6 | 54.0 | 53.1 | 43.8 | 65.0 | 45.8 | 0.38 |
| Nausea | 50.8 | 50.5 | 18.5 | 57.1 | 58.0 | 58.0 | 37.5 | 45.0 | 50.0 | 0.03 |
| Constipation | 25.9 | 36.6 | 25.9 | 23.8 | 12.0 | 27.2 | 18.8 | 22.5 | 33.3 | 0.11 |
| Insomnia | 21.2 | 25.8 | 22.2 | 23.8 | 16.0 | 21.0 | 12.5 | 15.0 | 20.8 | 0.81 |
| Diarrhea | 19.6 | 23.7 | 25.9 | 33.3 | 22.0 | 13.6 | 6.2 | 15.0 | 16.7 | 0.28 |
| Irritable Bowel Syndrome | 7.9 | 9.7 | 7.4 | 4.8 | 12.0 | 8.6 | 0.0 | 5.0 | 8.3 | 0.83 |
| None | 6.9 | 5.4 | 11.1 | 19.0 | 2.0 | 3.7 | 18.8 | 5.0 | 12.5 | 0.05 |

Chronic symptoms were self-reported among participants with POTS and were analyzed via ANOVA among phenotypes. Using Bonferroni correction, statistical significance was calculated to be p < 0.0025. There were no chronic symptoms that were statistically significant among phenotypes.

† reported in free text under “Other” at a frequency of > 4.0%

‡ reported in free text under “Other” at a frequency of < 4.0% included multiple infections ‖, fevers, swelling of the legs or feet, atopy ¶, hoarseness, slurred speech, sense of imbalance, enlarged lymph nodes, leg cramping when walking, easy bruising, multiple sensitivities^#^, dry eyes, and oral ulcers

§ Urinary symptoms included bladder spasms, urgency, leaking, weak urinary stream, hematuria, difficulty starting urination, loss of bladder control, urinary reflux, and hypercalciuria

‖ Multiple infections included: urinary tract infections, streptococcus infections, sinus infections, ear infections, bronchial infections, pneumonia, and viral infections

¶ Atopy included asthma, allergies, allergic reactions, hives, eczema, reactive airway disease, food allergies, and angioedema

^#^ Multiple sensitivities included light, sound, smell, foods, medications

A = Hyperadrenergic, N = Neuropathic, O = Hypovolemic

**Supplementary Table S4. Head Up Tilt Test (HUTT)**

|  |  | **All** | **Pure A** | **Pure N** | **Pure O** | **AN** | **AO** | **NO** | **ANO** | **None** |
| --- | --- | --- | --- | --- | --- | --- | --- | --- | --- | --- |
| Sample Size | | 378 | 93 | 27 | 21 | 50 | 81 | 16 | 40 | 24 |
| HR | |  |  |  |  |  |  |  |  |  |
|  | Supine | 75.7 ± 13.8 | 74.6 ± 12.4 | 74.1 ± 16.1 | 76.2 ± 11.6 | 78.4 ± 14.2 | 77.9 ± 16.0 | 69.6 ± 11.1 | 77.0 ± 13.6 | 69.7 ± 11.4 |
|  | Max | 109.2 ± 18.7 | 110.0 ± 18.5 | 104.2 ± 16.3 | 107.8 ± 20.1 | 110.6 ± 18.2 | 112.6 ± 21.0 | 97.7 ± 16.2 | 111.5 ± 17.5 | 105.0 ± 15.4 |
|  | Change | 33.5 ± 14.4 | 35.3 ± 14.5 | 30.1 ± 10.6 | 31.6 ± 16.4 | 32.2 ± 13.3 | 34.6 ± 12.3 | 28.1 ± 10.5 | 34.6 ± 12.3 | 35.3 ± 15.0 |
| SBP | |  |  |  |  |  |  |  |  |  |
|  | Supine | 110.6 ± 10.9 | 112.2 ± 9.7 | 110.9 ± 10.3 | 111.3 ± 10.3 | 109.9 ± 12.7 | 110.0 ± 12.5 | 108.6 ± 11.3 | 108.5 ± 11.1 | 111.3 ± 8.0 |
|  | Max | 115.4 ± 11.7 | 119.7 ± 11.2 | 113.5 ± 10.4 | 112.9 ± 10.2 | 115.2 ± 12.2 | 115.2 ± 14.0 | 110.0 ± 10.6 | 113.9 ± 10.6 | 112.0 ± 7.8 |
|  | Min | 103.1 ± 12.6 | 106.6 ± 11.6 | 102.0 ± 11.0 | 100.9 ± 11.2 | 103.4 ± 13.1 | 102.3 ± 15.1 | 99.8 ± 9.2 | 101.6 ± 12.4 | 100.6 ± 8.7 |
| DBP | |  |  |  |  |  |  |  |  |  |
|  | Supine | 67.4 ± 7.8 | 67.3 ± 7.4 | 68.4 ± 8.0 | 67.7 ± 7.0 | 67.2 ± 8.2 | 66.4 ± 8.3 | 67.2 ± 8.4 | 66.8 ± 8.7 | 69.2 ± 6.5 |
|  | Max | 76.6 ± 8.7 | 79.4 ± 8.2 | 73.6 ± 8.6 | 71.6 ± 6.3 | 78.2 ± 9.1 | 77.1 ± 9.3 | 71.1 ± 7.7 | 77.7 ± 8.5 | 73.1 ± 6.2 |
|  | Min | 65.2 ± 7.7 | 66.0 ± 7.1 | 66.7 ± 7.8 | 63.1 ± 7.0 | 65.4 ± 7.4 | 64.3 ± 8.2 | 64.5 ± 7.4 | 65.3 ± 9.0 | 65.1 ± 7.4 |

Means and standard deviations by phenotype of heart rate, systolic blood pressure, and diastolic blood pressure obtained from the HUTT.

HR = Heart Rate, reported in beats-per-minute

SBP = Systolic Blood Pressure, reported in mmHg

DBP = Diastolic Blood Pressure, reported in mmHg

Max = Maximum

Min = Minimum

A = Hyperadrenergic, N = Neuropathic, O = Hypovolemic

**Supplementary Table S5. Sheehan Disability Scale**

|  | **All** | **Pure A** | **Pure N** | **Pure O** | **AN** | **AO** | **NO** | **ANO** | **None** | **P-value** |
| --- | --- | --- | --- | --- | --- | --- | --- | --- | --- | --- |
| Sample Size | 378 | 93 | 27 | 21 | 50 | 81 | 6 | 40 | 24 |  |
| Work | 7.1 ± 2.8 | 7.0 ± 2.8 | 7.2 ± 3.0 | 7.0 ± 2.9 | 7.6 ± 2.3 | 7.2 ± 2.7 | 6.7 ± 3.0 | 7.3 ± 2.9 | 6.5 ± 3.3 | 0.85 |
| Social | 7.0 ± 2.5 | 6.8 ± 2.3 | 7.0 ± 2.3 | 6.2 ± 3.0 | 7.6 ± 2.1 | 6.9 ± 2.7 | 7.4 ± 2.5 | 7.7 ± 2.0 | 6.2 ± 3.3 | 0.13 |
| Family | 6.4 ± 2.8 | 6.2 ± 2.9 | 6.6 ± 2.8 | 5.5 ± 3.1 | 7.0 ± 2.5 | 6.3 ± 2.9 | 6.8 ± 2.7 | 7.3 ± 2.3 | 5.9 ± 3.2 | 0.16 |
| Days Lost | 3.0 ± 2.8 | 2.6 ± 2.8 | 2.8 ± 2.7 | 2.9 ± 2.9 | 3.5 ± 2.6 | 2.9 ± 2.7 | 3.2 ± 2.8 | 4.3 ± 2.5 | 2.8 ± 2.9 | 0.08 |
| Days Unproductive | 4.7 ± 2.3 | 4.4 ± 2.3 | 4.8 ± 2.4 | 3.8 ± 2.9 | 5.0 ± 2.3 | 4.9 ± 2.2 | 5.0 ± 2.2 | 5.2 ± 2.1 | 4.7 ± 2.3 | 0.26 |

Functional impairment was measured by the Sheehan Disability Scale, with scores compared among phenotypes. Using Bonferroni correction, statistical significance was calculated to be p < 0.01. There were no statistically significant differences in the scores among phenotypes
